# Mutations of familial hemophagocytic lymphohistiocytosis (FHL) related genes and abnormalities of cytotoxicity function tests in patients with macrophage activation syndrome (MAS) occurring in systemic juvenile idiopathic arthritis (sJIA)

**DOI:** 10.1186/1546-0096-12-S1-P53

**Published:** 2014-09-17

**Authors:** Claudia Bracaglia, Elena Sieni, Martina Da Ros, Carmela De Fusco, Concetta Micalizzi, Valentina Cetica, Benedetta Ciambotti, Maria Luisa Coniglio, Antonella Insalaco, Fabrizio De Benedetti, Maurizio Aricò

**Affiliations:** 1 Division of Rheumatology, Department of Pediatric Medicine, IRCCS Ospedale Pediatrico Bambino Gesù, Rome, Italy; 2 Department of Pediatric Hematology-Oncology, Meyer Children Hospital, Florence, Italy; 3 Department of Pediatric Hematology-Oncology, Pausillipon Children Hospital, Naple, Italy; 4 Department of Pediatric Hematology-Oncology, G. Gaslini Children Hospital, Genoa, Italy; 5 Istituto Toscano Tumori (I.T.T.), Florence, Italy

## Introduction

MAS is a severe complication of rheumatic diseases, mostly sJIA. Clinical and laboratory features are similar to those of FHL resulting from mutations in selected genes involved in the cytotoxicity pathway.

## Objectives

We investigated the presence of mutations of FHL-related genes and of abnormalities in degranulation and perforin expression, in patients with MAS occurring in the context of sJIA.

## Methods

From the HLH Italian National Registry, we selected patients with MAS defined according to the HLH 2004 criteria and with confirmed diagnosis of sJIA based on ILAR criteria. Mutation analysis was performed by Sanger sequencing of FHL-related genes. Perforin expression and degranulation were analyzed using flow-cytometry.

## Results

We identified 31patients (17 females; 25 Southern European, 6 Indian) with MAS and sJIA. Eleven patients (35.5%) had 14 monoallelic mutations in *PRF1* (n=7), *UNC13D* (n=1), *STX11* (n=1), *STXBP2* (n=4), and *Rab27a* (n=1). Three patients had mutations in 2 genes. Both degranulation and perforin expression were evaluated in 18 patients. At least one test was defective in 11 patients (61%). The clinical and laboratory features of patients with monoallelic mutation and/or with abnormalities in at least one functional test, were not different from those of the remaining patients. However, re-occurrence of MAS tended to be more frequent in patients carrying mutations (mutated 27% versus non-mutated 10%) and in patients showing abnormalities in at least 1 functional test (abnormal 18% versus 0%). One patient died of MAS: she carried the N252S *PRF1* variant and showed reduced perforin expression.

## Conclusion

Monoallelic mutations in FHL-related genes and partial defect in either perforin expression or degranulation capacity are frequently observed in patients with sJIA who develop MAS. Additional genetic studies are warranted to identify additional genes potentially linked to MAS development.

## Disclosure of interest

None declared.

